# Metabonomics on *Candida albicans* indicate the excessive H3K56ac is involved in the antifungal activity of Shikonin

**DOI:** 10.1080/22221751.2019.1657362

**Published:** 2019-08-27

**Authors:** ZeBin Liao, ZhenYu Zhu, Ling Li, Liang Wang, Hui Wang, YuanYing Jiang, YingYing Cao

**Affiliations:** a Department of Radiation Medicine, Faculty of Naval Medicine, Second Military Medical University, Shanghai, People’s Republic of China; b Shanghai Skin Disease Hospital, Tongji University School of Medicine, Shanghai, People’s Republic of China; c School of Pharmacy, Second Military Medical University, Shanghai, People’s Republic of China; d Department of Pharmacology, Shanghai Tenth People’s Hospital, Tongji University School of Medicine, Shanghai, People’s Republic of China

**Keywords:** Metabonomics, *Candida albicans*, Shikonin, hyperacetylation of the histone H3K56, *HST3*

## Abstract

Development of antifungal agents with novel mechanism and low toxicity are essential due to the prevalence of the infectious diseases caused by *Candida albicans*. The current study employed a new research method, which combined the ultra-high performance liquid chromatography with quadrupole time-of-flight mass spectrometry and gas chromatography-mass spectrometry, to investigate the intrinsic mechanism of Shikonin (SK) against *C. albicans*. The levels of 27 metabolites, which mainly involved in histone deacetylation, amino acid synthesis, lipid synthesis, nitrogen metabolism, tricarboxylic acid cycle, oxidative stress and glycolysis, were remarkably changed upon SK treatment. Specially, the down-regulation of nicotinamide (NAM) upon SK treatment indicated the suppression of the deacetylation of the histone H3 on lysine 56 residue (H3K56). Further experiment confirmed that the level of H3K56 acetylation (H3K56ac) was dramatically increased upon SK treatment which was mediated by *HST3*, the gene encoding the H3K56 deacetylase (Hst3p). Our results demonstrated that SK is the first natural compound reported to execute antifungal activity directly via boosting H3K56ac mediated by *HST3*. Importantly, this finding shed new light on the mechanisms to relieve the side effects or reverse the drug tolerance, as well as the development of agents for antifungal therapies.

## Introduction

Invasive infections caused by *Candida* species have emerged as an increasing challenge for clinicians. *C. albicans*, which is the symbiotic microbe in gastrointestinal tract, is the most common opportunistic pathogen causing fungal infectious disease [1,2]. Despite the continual effort to explore antifungals, available agents for anti-epiphyte therapy are still limited. Azoles, echinocandins and polyenes, which respectively target cytochrome-P450-dependent lanosterol 14-*α*-demethylase, *β*-1,3-glucan synthase and membrane sterols, are commonly employed in clinical setting*.* Nevertheless, the emergence of azole-resistant strains has been reported increasingly due to the prolonged use of these drugs [3]. In addition, the severe side effects of polyenes (e.g. Amphotericin B) [4], the high protein binding capacity and anaphylactic reaction of echinocandins (e.g. caspofungin) [5], the high cost of these drugs, as well as the intrinsic resistance of some pathogenic *Candida* species to antifungals [6,7] all bring great challenges to candidiasis treatment. The development of antifungals with novel mechanisms is strongly needed for medical practice.

Shikonin (SK) is a naphthoquinone pigment extracted from the rhizome of *Lithospermum erythrorhizon* [8]. In japan market, there is an ointment called “Shiun-ko” prepared by Shikon which is used to treat wounds, burn and hemorrhoids [9]. Like its genitor plant, SK has been claimed to exhibit multiple pharmacological properties including antiviral [10], antitumor [11,12], antidiabetic [13], antithrombotic, anti-inflammatory [14] and antifungal effects [9]. Previous study has shown that SK is a potent antifungal agent against various kinds of fungi, including *Candida krusei*, *Saccharomyces cerevisiae* and *Candida glabrata* [9]. Specially, the azole-resistant *C. albicans* strains were more susceptive to SK than fluconazole [15,16]. Nevertheless, the intrinsic mechanisms of SK against *C. albicans* remain to be fully demonstrated.

Generally defined as “the quantitative measurement of the dynamic multiparametric response of a living system to pathophysiological stimuli or genetic manipulations”, metabonomics is a useful tool widely used in microbiology, phytology and disease diagnosis [17,18]. When combined use with other “omic” studies, metabonomics is a practical tool for investigating the enormous metabolic diversity [19]. In the present study, we determined the metabolic profiles of SK-treated *C. albicans* by gas chromatography-mass spectrometry (GC/MS) and quadrupole time-of-flight tandem mass spectrometry (UHPLC-Q-TOF/MS) analysis, in which a depiction of the metabolic fingerprinting and metabolites changes between the SK-treated and untreated *C. albicans* cells were obtained. Specially, the decreased level of nicotinamide (NAM) upon SK treatment indicated the inhibition of lysine 56 on histone H3 (H3K56) deacetylation [20]. Besides, SK was found to induce hyperacetylation of H3K56 mediated by *HST3*. Our study provides a promising recipe for the development of antifungal agents with low toxicity and novel mechanism.

## Materials and methods

### Strains and compounds

Strains used in this study are listed as follows: *C. albicans* SC5314, *C. albicans* CASS1 and the *HST3* mutant strain *hst3*▵*/pTET-HST3*. SK, NAM, doxycycline (doxy) and *α*-aminobutyric acid were all purchased from Sigma-Aldrich Co. LLC (St. Louis, MO, US). During the experiments, 20 mg/ml doxy, 6.4 mg/ml SK as well as 200 mM *α*-aminobutyric acid in dimethyl sulfoxide (DMSO), and 200 mg/ml NAM in ultra-pure distilled water were used as stocks. The required concentrations of these drugs were obtained by diluting with culture suspensions.

### Cell culture and IC_50_ determination

*C. albicans* strains from Sabouraud dextrose agar were propagated in liquid YPD (1% w/v yeast extract, 2% w/v peptone, and 2% w/v dextrose) medium overnight at 30°C and 200 rpm. Cells were then suspended in yeast nitrogen base (YNB) (Difco) medium supplemented with 50 mM glucose to an optical density at 600 nm (OD_600_) of 0.1 and recovered from stationary phase at 30°C until an OD_600_ of 0.2 was reached. For the determination of the 50% inhibitory concentration (IC_50_) of SK, solution was treated with SK in a series of concentrations. IC_50_ was determined by measuring OD_600_ followed by cultivating the cultures for 4 h.

### Drug treatment and extraction of intracellular metabolites

Cells (OD_600 _= 0.2) were treated with 8 μg/ml SK or DMSO for 4 h. Metabolites of the cells were acquired as previously described with moderate modifications [21,22]. In brief, cells were washed rapidly with precooled ultrapure water in less than 1 min. Then the samples were frozen to −80°C followed by resuspending them in 1 ml boiling water (containing 10 μl of 200 mM *α*-aminobutyric acid as internal standard) for 15 min. The solution was processed by freeze−thaw cycles for 15 min at −80 and 60°C alternatively for 3 repeated times. The filtrate was freeze-dried after centrifugation, filtration and prefrozen in turn.

### Biomass dry weight measurement

At the final stage of the cultivation period, samples were filtered with a 0.45 μm filter followed by being washed with ultrapure sterilized water for three times. After air drying at room temperature, the specimens were measured at least three times to get a constant weight.

### Sample derivatization for GC-MS analysis

Samples were dried in a freeze-dryer overnight to get the freeze-dried powder. For derivatization, the samples were treated with 75 μl O-ethylhydroxylamine hydrochloride in pyridine (20 mg/ml) at 40°C for 90 min, after which 75 μl N-methyl-N-(trimethylsilyl)-trifluoroacetamide (MSTFA) was added and samples were cultivated at 40°C for 50 min. At the end of the cultivation stage, samples were centrifuged at 16100 ×* g* to collect the supernatant which then mixed with 100 μl heptane.

### Gas chromatography−mass spectrometry analysis

- The conditions of Gas chromatography spectrometry: Thermo Trace Ultra/DSQ II GC/MS (Thermo Electron Corporation) equipped with a HP-5MS capillary column (30 m × 0.25 mm × 0.25 μm) was employed. Temperatures were programmed as follows: 70°C for 3 min and increased by 4°C min^−1^ to 220°C followed by increasing to 310°C by 8°C min^−1^ and held for 10 min. Samples were collected at 7.6 min and lasted for 52 min. Helium was employed as the carrier at a flow rate of 1 ml/min without shunting.
- The conditions of mass spectrometry: Electron impact (EI) in the full scan mode (m/z 15−800) at 5 s/dec was employed. The interface temperature, ion source temperature and electron energy were set to be 200, 280°C and 70 eV, respectively. Data were processed with Xculibur system.

### Data processing

The data processing was operated as previous described [23]. Briefly, the data in RAW format were converted to CDF format files that were then nonlinear aligned and integrated by XCMS software (bw = 5, fwhm = 4 and snthersh = 5) to get a 3D matrix. The ion peaks were filtered with the MATLAB 7.0 (The MathWorks, Inc., USA) to eliminate the signal interference of fragment ions derived from the same endogenous metabolites, after which the ion peaks with the maximum kurtosis at the same retention time (the time bin is 0.01 min) were collected. To calibrate the mass spectrum response, the ratio of peak area of each sample and their biomass dry weight was calculated, and the output data was divided by the most abundance fragment ion for the silylation derivative of *α*-aminobutyrate (m/z 130.08). After the treatment above, data were preprocessed by par-scaling with SIMCA-P V 11.0 (Umetrics, Sweden), and then analyzed with principal components analysis (PCA), partial least-squares discriminant analysis (PLS-DA), variable influence in projection (VIP) and Student’s *t*-test (*P* < 0.01) in turn. Finally, the significant data were matched with the included NIST database of GC-MS to find the potential biomarkers that were determined as described in Results section.

### Quadrupole time-of-flight tandem mass spectrometry analysis

To determine the metabolite 7, the UHPLC-Q-TOF/MS spectrometry analysis was performed as previously described [24]. Briefly, Agilent 1290 Infinity LC system assemble with Agilent 6530 Accurate Mass Quadrupole Time-of-Flight mass spectrometer (Agilent, USA) was employed. The mobile phase was of (A) acetonitrile with 0.1% formic acid and (B) water with 0.1% formic acid. The ion peaks were separated by an ACQUITY UPLC HSS T3 column (2.1 mm × 100 mm, 1.8 μm, Waters, Milford, MA) at 40°C. The samples were injected into UHPLC-Q-TOF/MS spectrometry with the mobile phase running at a flow rate of 0.4 ml/min. The solvent gradient began with 2% A for 2 min, converted to 95% A for 13 min, and then maintained at 95% A for 2 min. The positive mode was an electrospray ionization source (ESI). And the capillary voltage of positive mode, negative mode, drying gas, nebulizer pressure the fragmentor voltage and skimmer voltage were set to be 4, 3 kV, 11 l/min (350°C), 45 psi, 120 and 60 V, respectively. Data were collected in profile mode from 50 to 1100 m/z.

The metabolite 7 was positioned by standard substances NAM (1 μg/ml) and isonicotinamide (1 μg/ml). To measure the content of NAM, six concentrations of NAM in pure water were injected into UHPLC-Q-TOF/MS spectrometry, from which a standard curve was obtained. And the peak areas of samples were converted into the concentrations of NAM.

### Antifungal susceptibility testing

The drug-susceptibility experiment was carried out as described previously [5, 9], with some modifications. In brief, strains were diluted with fresh YPD medium to the desired cell density and then treated with SK alone or in combination in certain concentration gradient. After incubation, OD_600_ value was measured and a representative drug susceptibility curve was obtained. As for the checkerboard microdilution assay, *C. albicans* SC5314 cells in RPMI 1640 medium was adjusted to 10^3^ CFU/ml. The concentrations of the agents are listed as following: 0.05–25.6 mg/ml for NAM, 0.125–64 μg/ml for SK. MIC_80_ was determined after being incubated at 30°C for 48 h, in which the lowest concentration of the agents that inhibited growth by 80% compared with that of drug-free wells was considered as MIC_80_.

### Western Blotting

Whole-cell lysates were acquired as previously described with slight modifications [25]. In brief, cells in log-phase in YPD were treated or untreated with agents in an orbital shaker at 30°C for 3.5 h, followed by resuspended in whole-cell extract buffer [350 mM NaCl, 20 mM Hepes (pH7.0), 0.1% Tween-20,10% glycerol] combined with protease inhibitor. After resuspending with 1 ml whole-extract buffer, pellets were agitated on a bead beater and lysates were collected by centrifugation. Then, protein obtained from total cell lysates were subjected to 15% polyacrylamide SDS gels, blotted with rabbit anti-H3K56ac (1:2000; Abcam) or rabbit anti-H3 (1:4000; Abcam), and tested with the chemiluminescence method after probed with secondary antibodies.

### Statistical analysis

The output data were normalized using internal standard and dry weight of each sample before exported to multivariate data analysis. Data were log transformed to eliminate the unit error before multivariate analysis. Multivariate statistical analyses such as PCA and PLS-DA were performed with the SIMCA-P version 11.0 software (Umetrics AB, Umea, Sweden). A threshold of VIP >1 generated after PLS-DA processing was employed to identify the variables that significantly contributed to the clustering and discrimination. Student’s *t*-test (SPSS software) was used to determine the significance, in which *P* values less than 0.01 with a fold-change ≥1.5 were considered significant. The peak areas of SK treated metabolites (VIP >1) were successively normalized by internal standard and biomass dry weight and the relative signal intensities of all peaks were obtained. The peak area ratios of each metabolite in SK-treated and the control groups were visually compared by plotting histogram.

## Results

### Selection of SK-induced metabolites

**Figure 1.**
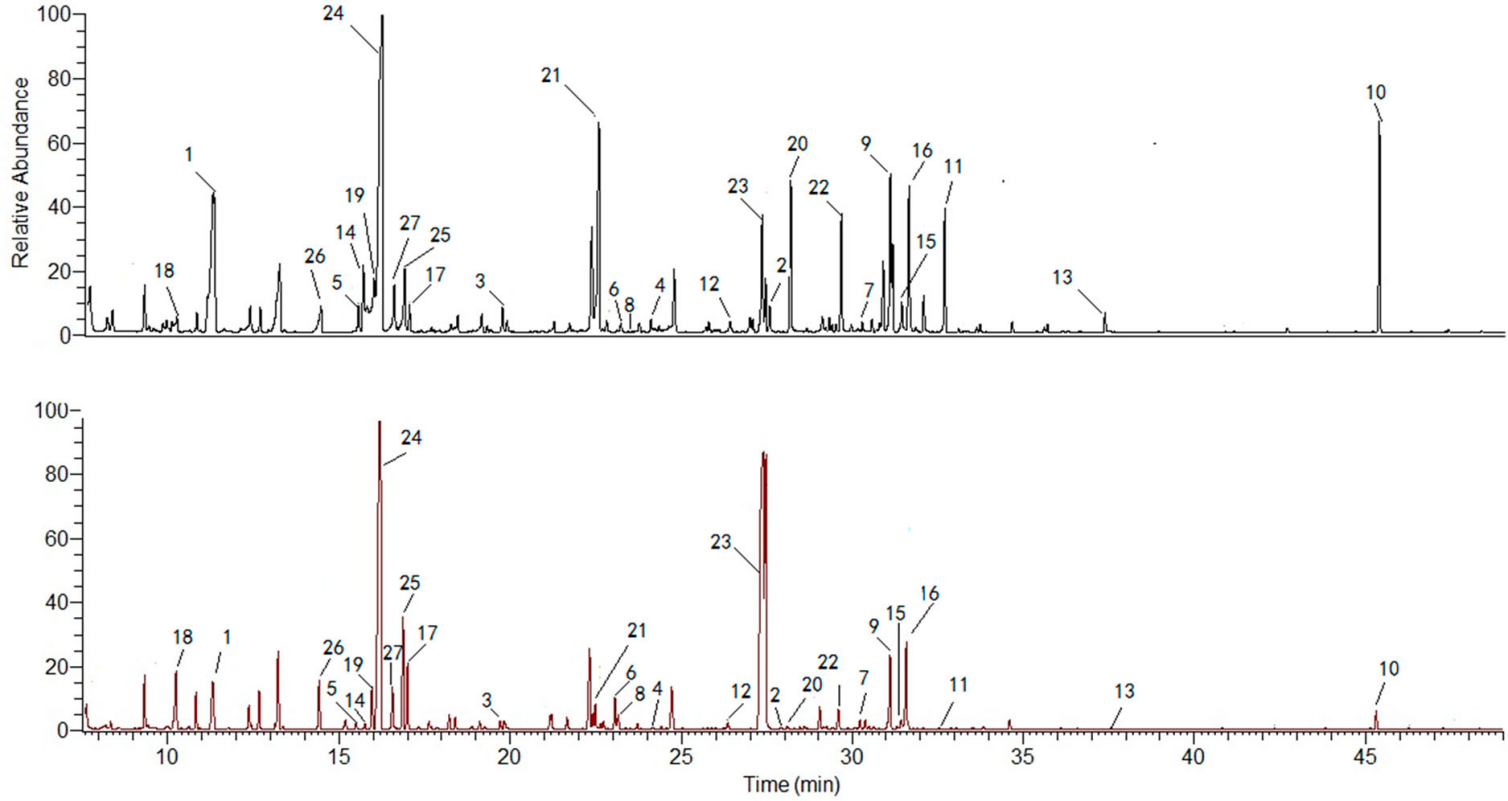
Typical GC/MS chromatograms of *C. albicans* SC5314 cells from the SK-treated group (A) or control group (B). 27 ion peaks were dramatically different between SK-treated group and control group. The related metabolites are listed in Table S1.

The differentially produced metabolites between the untreated and SK-treated (8 μg/ml) groups were tested. As the GC-MS characteristic chromatograms shown that, 6280 ion peaks were obtained via calibrating and integrating mass ions using XCMS software (Figure 1). To eliminate the interference of the same intracellular metabolites that were derived from silylation process, these fragment ion peaks were simplified by untargeted filtration and 1278 ion peaks were gained. After being processed by the unsupervised Principal Component Analysi, SK-treated and untreated groups were found to be separated clearly (*R*^2^ = 0.741) (Figure S1). To determine ion peaks between the groups discriminatingly, the PLS-DA method which works well in selecting biomarkers from similar metabolomics data [22] was employed. As shown in the first two components from the PLS-DA score plot, the discrimination between the control and SK-treated groups was confirmed (*R*^2^ *Y* = 0.956, *Q*^2^ = 0.878). As the model was validated with permutation testing generated the intercepts of *R*^2^ = 0.448 and *Q*^2^ = −0.186, the model was confirmed to have good prediction reliability and ability. The potential biomarkers were selected by the corresponding loading plot and S-plot (Figures S1C, D), in which the biomarkers were ascertained by the ions most distant away from the origin that contributed strikingly to the aggregation of the two groups. The S-plot, a filter helpful for identifying the vital metabolites in the model, presented the correlation and covariance between the model and the variables. The statistically significant metabolites contributing to the model were chosen via the variable influence in projection (VIP). VIP is generated from the PLS-DA processing, and 1 was the threshold value in our study.

94 variables were first selected as the candidates for biomarkers in terms of the approach mentioned above. Then, fewer variables were collected after the selfsame metabolites were merged to avoid the interference of variables derived from the same metabolites. To precisely select potential biomarkers for further study, *P*-values less than 0.01 with a fold-change ≥1.5 were set to be significant. Finally, the significant data was matched with the included NIST database of GC-MS and 27 metabolites were ascertained as the discrimination of SK-treated and the control groups in accordance with the data from GC-MS.

### Reduced level of NAM Production upon SK treatment

**Figure 2.**
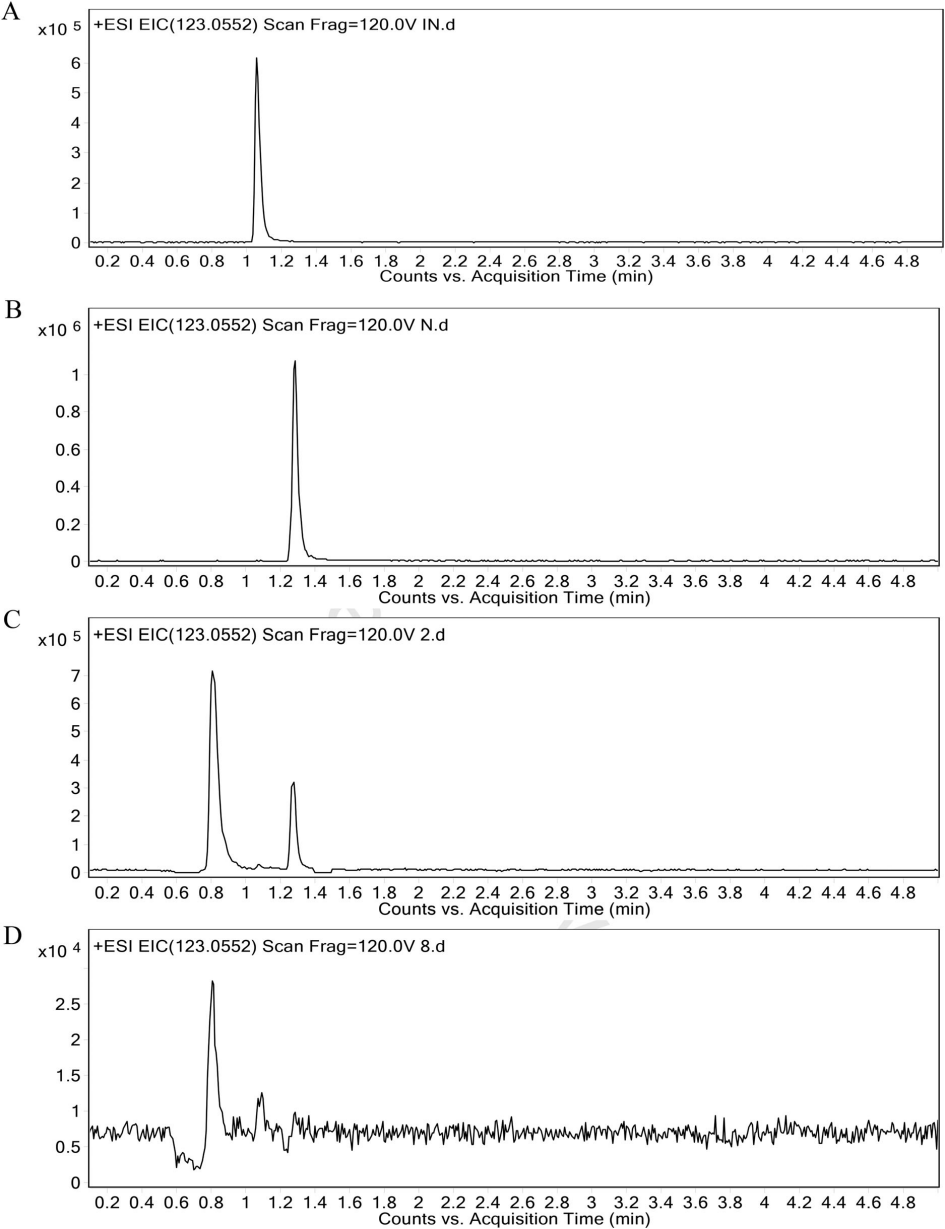
Typical UHPLC-Q-TOF/MS chromatograms of INAM (A), NAM (B), *C. albicans* SC5314 cells from the untreated (C) or SK-treated group (D). The retention time of INAM and NAM were respectively 1.06 and 1.28 min in UHPLC-Q-TOF/MS chromatogram. INAM, isonicotinamide; NAM, nicotinamide.

Because the retention time of INAM and NAM are so closer, the ion peaks of them in the GC/MS chromatograms are too closer to be distinguished. Thus, UHPLC-Q-TOF/MS, which offers plentiful fragmentation ion information and exact mass measurement [26], was employed to identify the metabolite. By utilizing standard substances, we found that INAM and NAM were separately eluted at 1.06 and 1.28 min in UHPLC-Q-TOF/MS chromatogram (Figure 2(A,B)). However, as is shown in the chromatograms of *C. albicans*, there was an apparent ion peak at 1.28 min but not at 1.06 min in the untreated group, while the peak disappeared after the treatment of SK (Figure 2(C,D)). To measure the content of NAM, a standard curve (*R*^2^ = 0.9998) was obtained (Figures S2). The result showed that, while 12.852 μg/g NAM was quantified in the untreated group on average, it could not be detected after SK treatment under the design condition, in which the limit of detection (LOD) in the experiment condition was found to be 3.4 pg (Table 1). Therefore, it was NAM but not INAM that was reduced in the metabolic profiles of *C. albicans* cells treated by SK.

**Table 1.** Nicotinamide content (μg/g) in the SK-treated or untreated group.

	1	2	3	4	5	6	average value
Control group	17.64	13.58	11.71	12.07	11.29	10.83	12.85
SK-treated group	<LOD^a^	<LOD	<LOD	<LOD	<LOD	<LOD	**–**

^a^LOD, limit of detection.

### Levels of 27 metabolites were remarkably changed upon SK treatment

**Figure 3.**
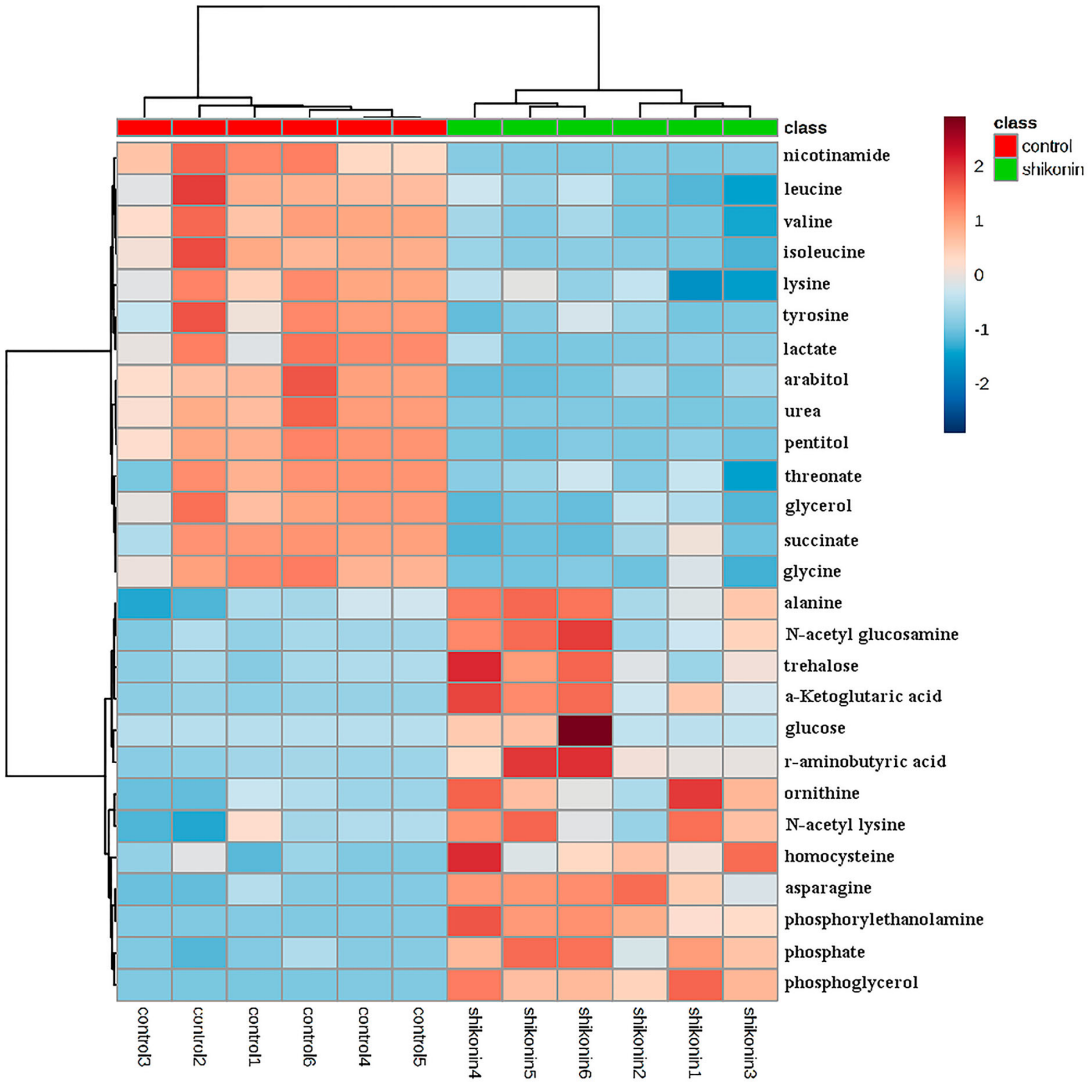
Colours in metabolite arrays represent *x*-fold change in the metabolite concentration in SK-treated and control groups. Brick-red indicates increased concentration levels of metabolites; blue indicates decreased concentration levels of metabolites. The two groups can be well recognized by cluster analysis of 27 potential biomarkers.

Based on the aforesaid experiments, 27 metabolites which might distinguish between SK-treated and the control groups were ascertained. The levels of them were shown in Figure S3 and Figure 3, and the descriptions of these metabolites, including their presumptive biological function and relatively changed levels, were shown in Table S1. Among the metabolites determined above, 13 out of 27 including phosphate, phosphoglycerol, γ-aminobutyrate, N-acetyl-lysine, alanine, ornithine, homocysteine, asparagine, N-acetyl-glucosamine, trehalose, glucose, *α*-ketoglutarate and phosphoethanolamine were increased in SK-treated group, while arabitol, glycine, glycerol, valine, isoleucine, leucine, lysine, tyrosine, succinate, lactate, urea, threonate, and pentitol were reduced in SK-treated group.

### Effect of NAM on the antifungal activity of SK

**Figure 4.**
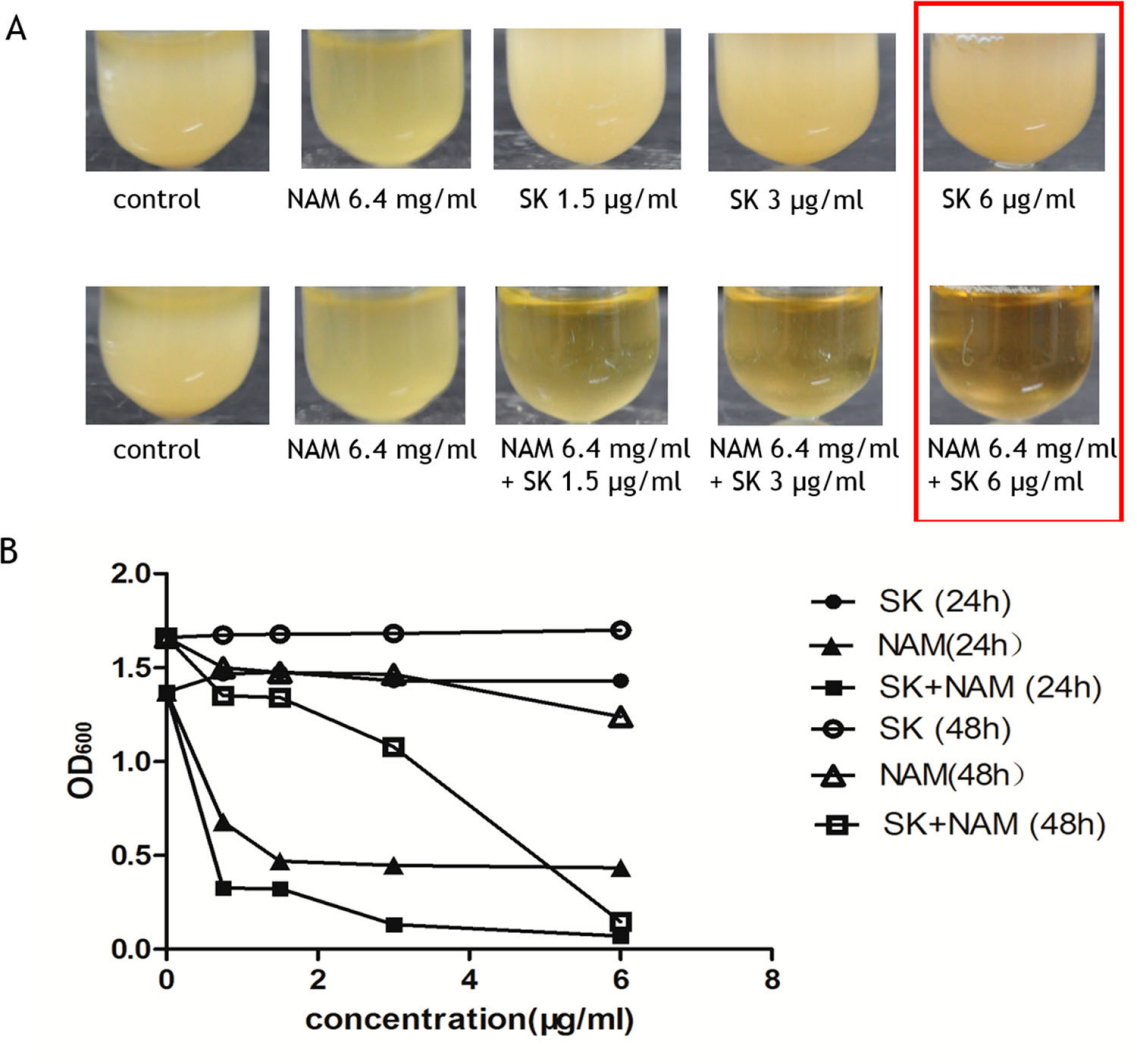
Effect of the combined use of NAM (6.4 mg/ml) and SK [(A) and (B)] on the growth of the strain *C. albicans* SC5314. *Candida* cells were incubated at 30°C for 24 h (filled symbols) or 48 h (open symbols), in which the samples were taken and measured the values of optical density at 600 nm (OD_600_). The photograph was taken after incubating for 24 h. NAM, nicotinamide; SK, Shikonin.

In *C. albicans*, NAM can selectively inhibit the deacetylation of histone H3 on lysine 56 (H3K56ac) reaction by suppressing the activity of histone deacetylase [20]. In view of the reduced level of NAM in SK-treated *C. albicans* cells, we first detected whether NAM could synergize the activity of SK. As is shown in the result, after incubating for 24 h in YPD medium, SK alone could not effectively inhibit the growth of *C. albicans* even when the dosage was up to 6 μg/ml (Figure 4(A,B)). However, when we added 6.4 mg/ml NAM, the antimycotic effect of SK was greatly enhanced even when its concentration was below 1 μg/ml (Figure 4(A,B)), and the inhibition was still noteworthy in 48 h (Figure 4(B)). Consistently, the synergistic effect between SK and NAM was observed with micro-broth dilution assay, in which the fractional inhibitory concentration (FIC) index was 0.38 (Table S2).

### SK Inhibits the deacetylation of histone H3 on lysine 56

**Figure 5.**
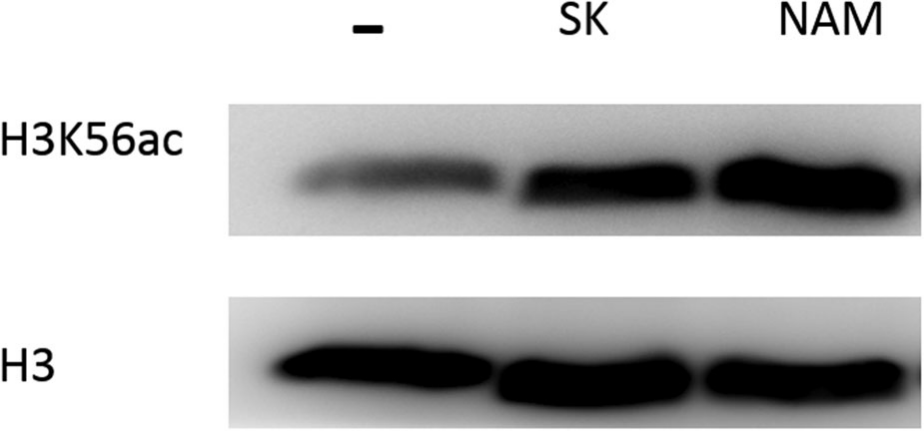
Immunoblot analysis of H3K56ac from whole-cell extracts of *C. albicans* SC5314 untreated, SK-treated (8 μg/ml), and NAM-treated (6.4 mg/ml). Protein was separated with SDS gels, and then probed with rabbit anti-H3K56ac or rabbit anti-H3 to detect the levels of H3K56ac and H3 protein. NAM, nicotinamide; SK, Shikonin.

NAM is the byproduct of the deacetylation process of histone H3 on lysine 56 (H3K56) in cells [20, 27], and the decreased level of NAM after SK treatment indicated the poor deacetylation activity. We next determined whether the antifungal activity of SK was involved in the H3K56ac with immunoblotting. As is shown in Figure 5, the H3K56ac level in *C. albicans* was strikingly increased by the treatment of 8 μg/ml SK, which was consistent with the effect by NAM (the concentration of NAM is 6.4 mg/ml).

### The hyperacetylation of H3K56 by SK was mediated by *HST3*

**Figure 6.**
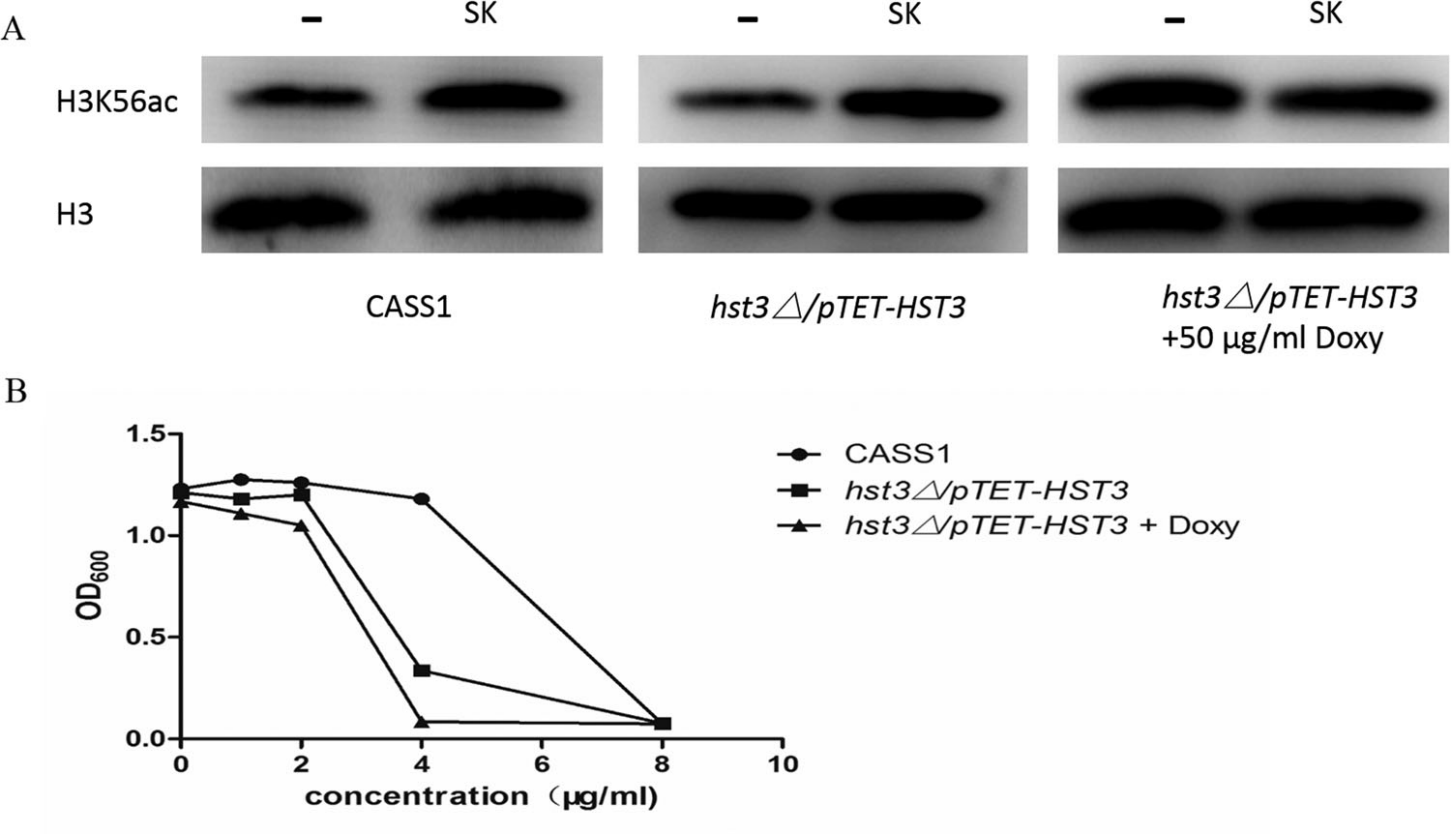
(A) Western blot of H3K56ac in wild-type (CASS1) or *hst3*▵*/pTET-HST3* (with/without doxycycline) cells upon SK treatment (8 μg/ml). Protein was separated with SDS gels, and then probed with rabbit anti-H3K56ac or rabbit anti-H3 to detect the levels of H3K56ac and H3 protein. (B) Sensitivity of *C. albicans* mutants to SK. Strains of the *HST3* mutant (*hst3*▵*/pTET-HST3*) with or without doxycycline, and the wild type were treated with SK. All strains were incubated at 30°C, in which the samples were taken and measured the values of optical density at 600 nm (OD_600_). SK, Shikonin; doxy, doxycycline.

In *C. albicans*, the H3K56 deacetylase (Hst3p) is encoded by the *HST3* gene, the *hst3Δ/pTET-HST3* strain, in which a single allele of *HST3* is deleted and the other allele can be inhibited by a promoter under the regulation of doxycycline (doxy), was used to explore the mechanism brought about the hyperacetylation of H3K56 in *C. albicans* induced by SK. As is shown in our results, the levels of H3K56ac in *C. albicans* CASS1 (wild type) and the *hst3Δ/pTET-HST3* strain were remarkably enhanced after the treatment of SK (8 μg/ml). However, the excessive level of H3K56ac could not be induced by SK when the other copy of the *HST3* gene was repressed by 50 μg/ml doxy (Figure 6(A)). Congruously, the *C. albicans cells* became more susceptible to SK when one copy or both of the *HST3* genes were repressed (Figure 6(B)). Thus, the hyperacetylation of H3K56 by SK was mediated by *HST3*.

## Discussion

Fungal species have reported to acquire resistance against many useful antifungal drugs, and there is a need to develop new antifungal drugs with novel mechanisms. Our previous study have shown that SK exerted general antifungal activity against *C. albicans* isolates via the augmentation of reactive oxygen species and the accumulation of nitric oxide [15, 16]. In the current study, we explored the intrinsic mechanism of SK against *C. albicans* with the combined use of GC/MS and UHPLC-Q-TOF/MS analysis. The results showed that 27 metabolites were dramatically induced by SK in *C. albicans*. Special subclass of metabolites were involved in histone deacetylation, amino acid synthesis, oxidative stress, TCA cycle, cell membrane synthesis, etc.

### Deacetylation of H3K56

In *C. albicans*, as the ribose 2′-OH of NAD^+^ accept the acetyl group transferred from the targeted protein, free NAM and 2′-O-acetyl-ADP-ribose were released during the deacetylation process of histone H3 on lysine 56 (H3K56) [20,27]. Here, the dramatically decreased level of NAM upon SK treatment indicates the inhibition of the deacetylation of H3K56. Firstly, the effect of the histone deacetylases inhibitor NAM on the antimycotic activity of SK was tested to determine whether there was synergism between NAM and SK. Our results showed that NAM could significantly enhance the antifungal activity of SK. As NAM is a byproduct derived from the NAD^+^-dependent deacetylation reaction, the reduced level of NAM by SK treatment indicated the inhibition of the deacetylation process in *C. albicans* [20]. Besides, NAM is the only selective inhibitor reported to induce hyperacetylation of H3K56 by suppressing the activity of histone deacetylase [20,28–30]. The reduced level of NAM in SK-treated *C. albicans* and the enhanced antifungal effect of SK against *C. albicans* by NAM prompted us to assume that SK could simulate the activity of NAM, in which the excessive level of H3K56ac due to the deacetylation process blocking in *C. albicans* could be observed. As anticipated, the results showed that both SK and NAM could remarkably induce the excessive H3K56ac level in *C. albicans*. Given that the *HST3* encodes a deacetylase responsible for the deacetylation of H3K56ac in *C. albicans* [20], we hypothesized that SK-treated *C. albicans* cells should phenocopy *HST3* repression. Unsurprisingly, when both of *HST3* was inhibited, hyperacetylation of H3K56 could not be induced by SK in *C. albicans*. Consistently, SK executed better antifungal activity against *C. albicans* with *HST3* repression. Besides, the rtt109 mutant was not more susceptible to SK as compared to the wild type strain, while the enhanced effect of the addition of NAM was diminished (data not shown), these results further indicate that the hyperacetylation of H3K56 is essential for the antifungal activity of SK against *C. albicans*.

Found in the mitotic S phase and premeiotic throughout the genome, acetylation of H3K56 play an essential role in many cellular processes including chromatin assembly, DNA repair and transcription [20,31]. However, the excessive acetylation due to the blocking of H3K56 deacetylation is cytotoxic to cells, and our result demonstrated that the hyperacetylation of H3K56 was involved in the antifungal activity of SK against *C. albicans*. To the best of our knowledge, SK is the first natural compound reported to exert antifungal activity via inducing the excessive level of H3K56ac in *C. albicans*. Previous study have demonstrated that the fungal enzymes that regulate H3K56ac have diverged considerably from their human counterparts [32] and the synergy effect between SK and NAM were seen, the potential clinical application of SK, alone or in combination with NAM or future fungal-specific modulators of H3K56ac would be expected to be a promising strategy in treating fungal disease. Although further in vivo studies will be necessary to assess the true therapeutic potential of SK and NAM, our results constitute a proof of principle that this strategy represents a useful therapeutic avenue.

Interestingly, the transcription levels of genes related to the deacetylation of H3K56 (including *RTT109*, *HST3*, *ASF1* and *VPS75*) showed no remarkable difference between the control and SK-treated groups (data not shown). As previously mentioned, H3K56ac in *C. albicans*, which is the physiological substrate of Hst3p, is catalyzed by the H3K56 acetyltransferase (Rtt109p) encoded by *RTT109* gene. Besides, several histone chaperones essential to the specificity and catalytic activity of Rtt109p (including Vps75p and Asf1p) have been identified [33]. Our result indicates that SK might directly influence the proteins related to H3K56ac instead of the genes or mRNA encoding them, which remains further investigation.

### Other metabolites

Levels of various metabolites were changed upon SK treatment, which might be related to the metabolism of amino acids, oxidative stress, cell wall, lipid synthesis and energy metabolism. It is known that many nutrients, including amino acids are precursors of intermediates in the citric acid cycle (TCA cycle); since all amino acids can be synthesized by fungi, and the TCA cycle is vital in forming a key part of aerobic respiration in cells, the change levels of amino acids which are precursors of the TCA intermediates strongly indicate the disturbance of TCA cycle. More confirmatory evidence comes from the changed levels of the three critical intermediates of Kreb’s cycle metabolites, succinate, lactate and *α*-Ketoglutarate.

The redox homeostasis of fungal pathogens is maintained by the co-option of enzymatic and non-enzymatic mechanisms, which can also help cells defense oxidative stress [34]. Here, the changed levels of metabolites involved in oxidative stress were also observed, including trehalose, lysine, 1,4-diamhobutanone, ornithine, alanine and GABA. Previous study have shown that *C. albicans* lacking *RTT109* are more susceptible to oxidative stress [25], and the induction of ROS and RNS were reported in our previous work [15, 16], the crosstalk between oxidative stress and hyperacetylation of H3K56 induced by SK remains further investigation.

The reduced levels of arabitol, glycerol, pentitol, glycerinate, threonate, which are osmolytes or the related metabolites utilized by *C. albicans* to alleviate hypertonicity [35], was observed in SK-treated cells. Besides, the intracellular levels of GlcNAc and glucose, both of which are monomeric units of chitin and *β*-Glucan in cell wall, respectively, could be stimulated by SK. It is reasonable to hypothesize that the reduced levels of arabitol, glycerol, pentitol, glycerinate, threonate as well as certain metabolites mentioned above is due to the membrane leakage associated with cell wall integrity and osmotic equilibrium damage by SK.

Meanwhile, the increased levels of intracellular N-Acetylglycine, N-acetyl-lysine phosphate, phosphoglycerol and phosphoethanolamine might be related to the adaptive reaction for membrane leakage. Since these metabolites are also essential nutrients in lipid synthesis and energy metabolism, the increased levels of these components might be associated with the interference of SK in the corresponding pathways.

## Conclusion

**Figure 7.**
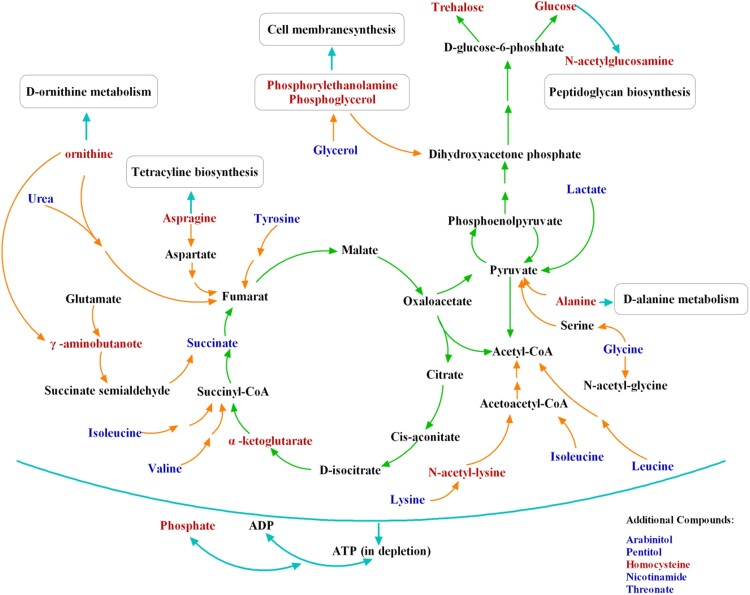
Schematic overview of differences in metabolite change between SK-treated and untreated *C. albicans* cells. The differently produced metabolites are mainly involved in tricarboxylic acid (TCA) cycle, amino acid synthesis, oxidative stress, nitrogen metabolism, lipid synthesis and glycolysis. Specially, the reduced level of nicotinamide indicated the deacetylation of H3K56 inhibition in *C. albicans* by SK, and the inhibition was mediated by *HST3* which then resulted in the excessive level of H3K56ac. Metabolites in red represent those metabolites that always remained at a high level after the exposure to SK; metabolites in blue represent those metabolites that were down-regulated after SK treatment. SK, shikonin.

Metabonomic on *C. albicans* presented here depicted the intrinsic mechanism in which SK executed its antifungal activity against *C. albicans* (Figure 7). Special subclass of metabolites was involved in amino acid synthesis, oxidative stress, TCA cycle, lipid synthesis, nitrogen metabolism and glycolysis. As a stress response, the levels of some osmolytes including arabitol, glycerol and pentitol were decreased, while trehalose biosynthesis was enhanced to defense against SK-induced oxidative injury. Specially, the decreased level of nicotinamide indicated the inhibition of H3K56ac deacetylation in *C. albicans* by SK, and this inhibition was mediated by *HST3* which then resulted in the excessive H3K56ac. As SK is the only natural compound reported to exert its antifungal activity via inhibiting the activity of histone deacetylase Hst3p, it provides a novel molecular skeleton for designing small molecular compound selectively inhibit the activity of fungal histone deacetylase, without affecting that of human beings. Besides, although quinones of the SK type possess side-effects, which include prooxidant effects and cytochrome P450 inhibition [36], it can be considered for topical applications, such as cutaneous fungal infection or vaginal infection. Together, our results demonstrated that SK can be used for the development of antifungal agents with low toxic and novel mechanisms, as well as shed new light on the mechanisms to relieve the side effects or reverse the drug tolerance in *C. albicans*.

## Supplementary Material

Supplemental Material
