# Supplementary figures and images for: Metabonomics on Candida albicans indicate the excessive H3K56ac is involved in the antifungal activity of Shikonin
Source: Emerg Microbes Infect. 2019 Aug 27;8(1):1243–53. doi: 10.1080/22221751.2019.1657362 (PMC6735332; doi:10.1080/22221751.2019.1657362)

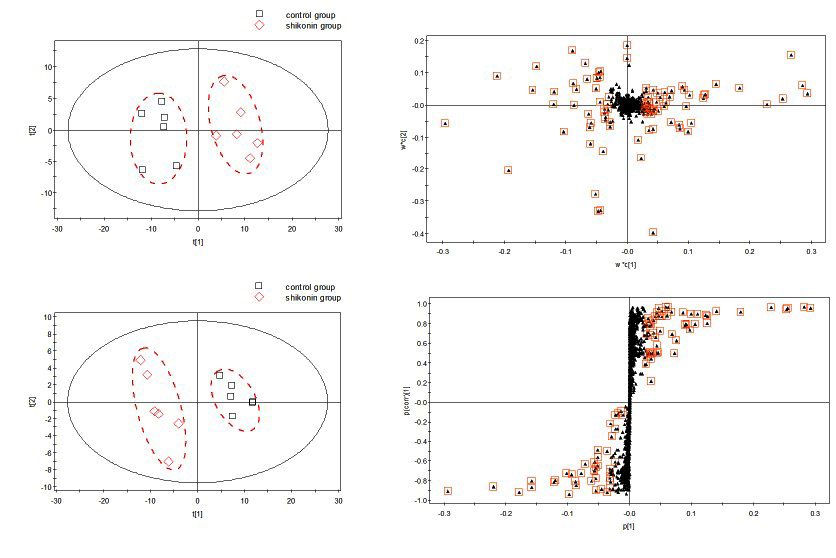

Supplement: Supplemental Material [file TEMI_A_1657362_SM7957.zip › Supplementary Figure S1 (new).tif]

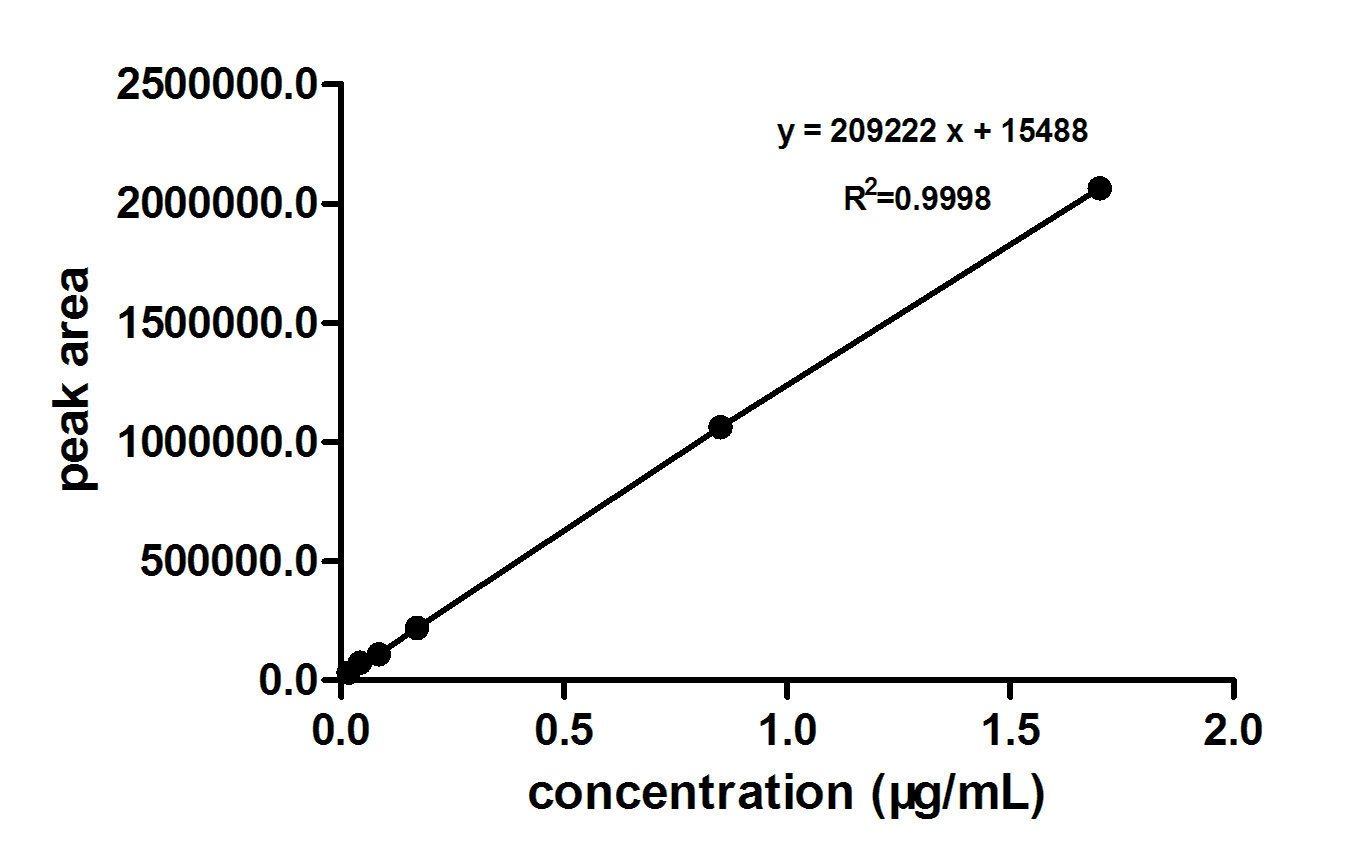

Supplement: Supplemental Material [file TEMI_A_1657362_SM7957.zip › Supplementary Figure S2 (new).tif]

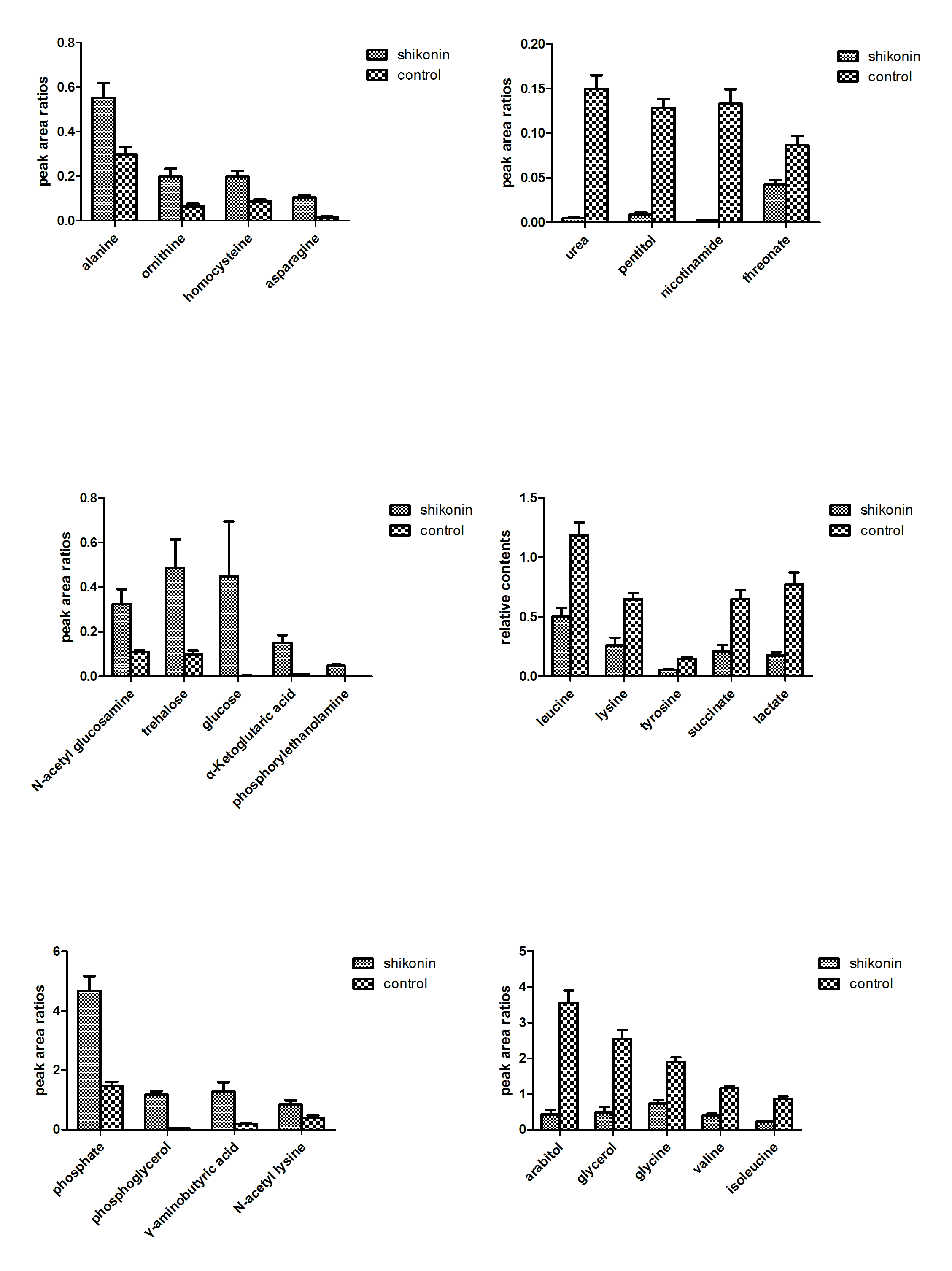

Supplement: Supplemental Material [file TEMI_A_1657362_SM7957.zip › Supplementary Figure S3 (new).tif]
