# Supplementary material for: Metabonomics on Candida albicans indicate the excessive H3K56ac is involved in the antifungal activity of Shikonin
Source: Emerg Microbes Infect. 2019 Aug 27;8(1):1243–53. doi: 10.1080/22221751.2019.1657362 (PMC6735332; doi:10.1080/22221751.2019.1657362)
Supplement: Supplemental Material [file TEMI_A_1657362_SM7957.zip › Supplementary Table S1 (new).doc]

| Supplementary Table S1 Metabolites with statistical differences between SK-treated and untreated groups. | | | | | |
| --- | --- | --- | --- | --- | --- |
| No. | RT(min)^a^ | Metabolite | Trend | Fold | Funtional Category |
| 1 | 11.28 | alanine | ↑ | 1.85 | amino acid metabolism |
| 2 | 27.5 | ornithine | ↑ | 3.04 | amino acid metabolism |
| 3 | 19.69 | homocysteine | ↑ | 2.3 | amino acid metabolism |
| 4 | 24.02 | asparagine | ↑ | 6.2 | amino acid metabolism |
| 5 | 15.19 | urea | ↓ | 30.24 | nitrogen metabolism |
| 6 | 23.09 | pentitol | ↓ | 14.33 | others |
| 7 | 30.39 | nicotinamide | ↓ | 65.59 | histone deacetylation |
| 8 | 23.15 | threonate | ↓ | 2.06 | others |
| 9 | 31.06 | N-acetyl-glucosamine | ↑ | 2.96 | glycolysis/gluconeogenesis |
| 10 | 45.32 | trehalose | ↑ | 4.86 | oxidative stress |
| 11 | 32.63 | glucose | ↑ | 144.7 | glycolysis/gluconeogenesis |
| 12 | 26.92 | α-Ketoglutarate | ↑ | 16.93 | TCA cycle |
| 13 | 37.31 | phosphoethanolamine | ↑ | 178.52 | lipid metabolism |
| 14 | 15.95 | leucine | ↓ | 0.42 | amino acid metabolism |
| 15 | 31.11 | lysine | ↓ | 2.48 | amino acid metabolism |
| 16 | 31.44 | tyrosine | ↓ | 2.78 | amino acid metabolism |
| 17 | 16.99 | succinate | ↓ | 3.09 | TCA cycle |
| 18 | 10.19 | lactate | ↓ | 4.39 | TCA cycle |
| 19 | 16.12 | phosphate | ↑ | 3.2 | energy metabolism |
| 20 | 28.1 | phosphoglycerol | ↑ | 32.52 | lipid metabolism |
| 21 | 22.48 | γ-aminobutyrate | ↑ | 6.85 | amino acid metabolism |
| 22 | 29.57 | N-acetyl-lysine | ↑ | 2.17 | amino acid metabolism |
| 23 | 27.33 | arabitol | ↓ | 8.32 | others |
| 24 | 16.16 | glycerol | ↓ | 5.25 | oxidative stress |
| 25 | 16.84 | glycine | ↓ | 2.6 | amino acid metabolism |
| 26 | 14.4 | valine | ↓ | 2.89 | amino acid metabolism |
| 27 | 16.54 | isoleucine | ↓ | 3.78 | amino acid metabolism |
| ^a^ RT, retention time. | | | | | |
|  |  |  |  |  |  |
