# Supplementary material for: Metabonomics on Candida albicans indicate the excessive H3K56ac is involved in the antifungal activity of Shikonin
Source: Emerg Microbes Infect. 2019 Aug 27;8(1):1243–53. doi: 10.1080/22221751.2019.1657362 (PMC6735332; doi:10.1080/22221751.2019.1657362)
Supplement: Supplemental Material [file TEMI_A_1657362_SM7957.zip › Supplementary Table S2 (new).doc]

| Supplementary Table S2 Interaction of Shikonin ( SK) and nicotinamide (NAM) against *C. albicans* SC5314 by microdilution assay. | | |  | |  | |  |  |  |
| --- | --- | --- | --- | --- | --- | --- | --- | --- | --- |
|  | 48 h MIC_80_ ^a^ | | |  | |  |  |  |  |
|  | SK | NAM | |  | |  |  |  |  |
| Antifungal agents alone | 8 | ≥25.6 | |  | |  |  |  |  |
| Combination with NAM | 2 | 3.2 | |  | |  |  |  |  |
| FIC index^b^ | 0.38 |  | |  | |  |  |  |  |
| ^a^ The unit of SK is μg/ml, while the unit of NAM is mg/ml; | | | | | | | | | |
| ^b^ Synergy and antagonism were defined by FIC indices of ≤0.5 and ＞ 4, respectively. An FIC index result of ＞0.5 but ≤ 4 was considered indifferent. | | | |  | |  |  |  |  |
